# Successful Endoscopic Removal of Toothpick Perforating Gastric Antrum With Over-the-Scope Padlock Clip Closure

**DOI:** 10.7759/cureus.11263

**Published:** 2020-10-30

**Authors:** Darshan Suthar, Elisabeth H Kramer, Harshit S Khara

**Affiliations:** 1 Division of Gastroenterology, Hepatology & Nutrition, Geisinger Medical Center, Danville, USA

**Keywords:** toothpick, gastrointestinal perforation, padlock clip, emergency gastroenterology and endoscopy, advance endoscopy, over-the-scope clip, foreign bodies, foreign body retrieval, device closure, gi endoscopy

## Abstract

Our unique case demonstrates the use of an over-the-scope Padlock clip for closure of a sharp foreign body induced gastric perforation, avoiding the need for surgical intervention. A 47-year old female presented with a two-day history of abdominal pain with nausea. Abdominal CT scan revealed a linear density in the distal body of the stomach extending outside the lumen. Endoscopic evaluation revealed a toothpick perforating through the wall of the gastric antrum. Endoscopic removal was performed, and closure of the full-thickness defect was achieved with an over-the-scope Padlock clip. The patient subsequently made an uneventful recovery, with no reported complications at two-year follow-up. Early endoscopic removal and closure of gastric luminal perforations by over-the-scope Padlock clips are viable alternative treatments for defects previously considered only amenable to surgical repair. Endoscopic treatment of gastrointestinal perforations has shown to decrease the morbidity and mortality associated with more invasive surgical procedures.

## Introduction

Toothpick ingestion is a relatively rare occurrence and often an accidental event related to food consumption. The most common locations of toothpicks prior to retrieval were small intestine (41%), large intestine (37%), stomach (20%), and esophagus (2%) [1]. Patients mainly present with abdominal pain, and complications may include gastrointestinal perforation, GI hemorrhage, peritonitis, sepsis and/or death.

Sharp-pointed objects may not be apparent on radiographic evaluation and it is recommended that these objects warrant urgent or emergent endoscopic removal depending on size and location [2,3]. Management of gastrointestinal perforations often necessitate surgical intervention; however, alternative endoscopic therapies such as self-expanding metal stents (SEMs), through-the-scope (TTS) clips, endoscopic suturing, and over-the-scope clip (OTSC) may be preferential over more invasive surgical options. SEMs often have a risk of migration and incomplete seals. TTS clips may not effectively close larger or full-thickness defects. Endoscopic suturing requires specialized equipment, is difficult in challenging locations, and may be limited by endoscopist experience [4,5]. OTSCs are particularly advantageous due to their safety, ease of use, and rapid deployment with full-thickness closure using standard endoscopes.

OTSCs are a category of novel clipping devices made of an elastic alloy, Nitinol, which has demonstrated clinical and technical success in the treatment of gastrointestinal hemorrhage, perforation, fistulae, anastomotic leaks, defect closure following endoscopic resection, and stent fixation [4]. The two types of OTSCs currently available are the bear-claw shaped Ovesco clip (Ovesco, Tübingen, Germany) and the more recent hexagonal-shaped Padlock clip (Steris, Mentor, OH, USA) with a full circumferential closing system [6]. Compared to commonly used endoscopic clips, the OTSCs can invaginate a larger volume of tissue with a greater mechanical compression force allowing for full-thickness closure of gastrointestinal perforations between 1 and 3 cm with a single clip application [4-8].

## Case presentation

**Figure 1:**
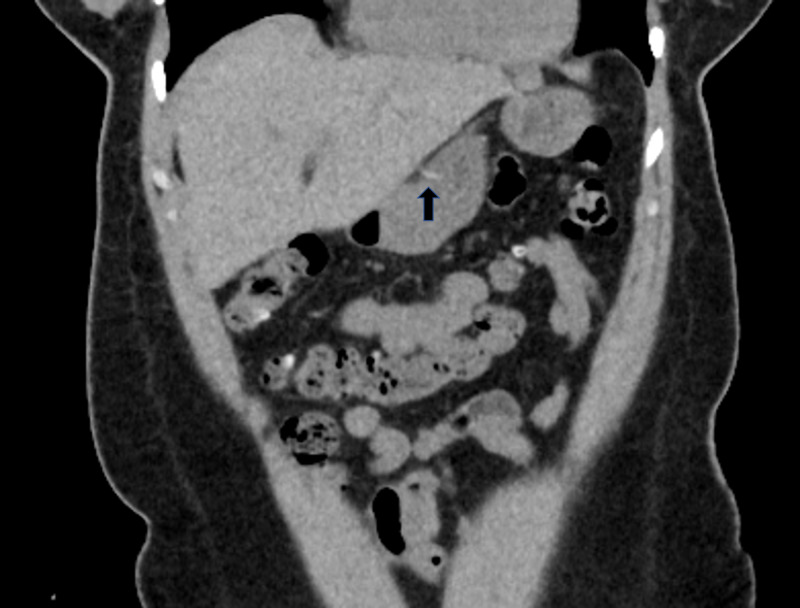
Abdominal CT scan revealing toothpick in distal body of stomach extending out of the lumen

A 47-year-old female with no significant past medical history presented with two days of progressively worsening sharp, intermittent, post-prandial abdominal pain associated with nausea. On examination, she was afebrile, hypertensive, and with diffuse abdominal tenderness, particularly in the epigastrium. She had a normal CBC, comprehensive metabolic panel and lipase. Initially, an abdominal ultrasound revealed a linear echogenicity traversing through the pylorus, possibly suggestive of a foreign body. Patient subsequently underwent abdominal x-ray which was unremarkable for foreign bodies. An abdominal CT scan revealed a 4 cm linear density in the distal body of the stomach which appeared to extend outside the lumen, without pneumoperitoneum (Figure 1). It was determined that the tip of the toothpick was not in contact with any other organ, but rather within the peritoneal cavity.

**Figure 2:**
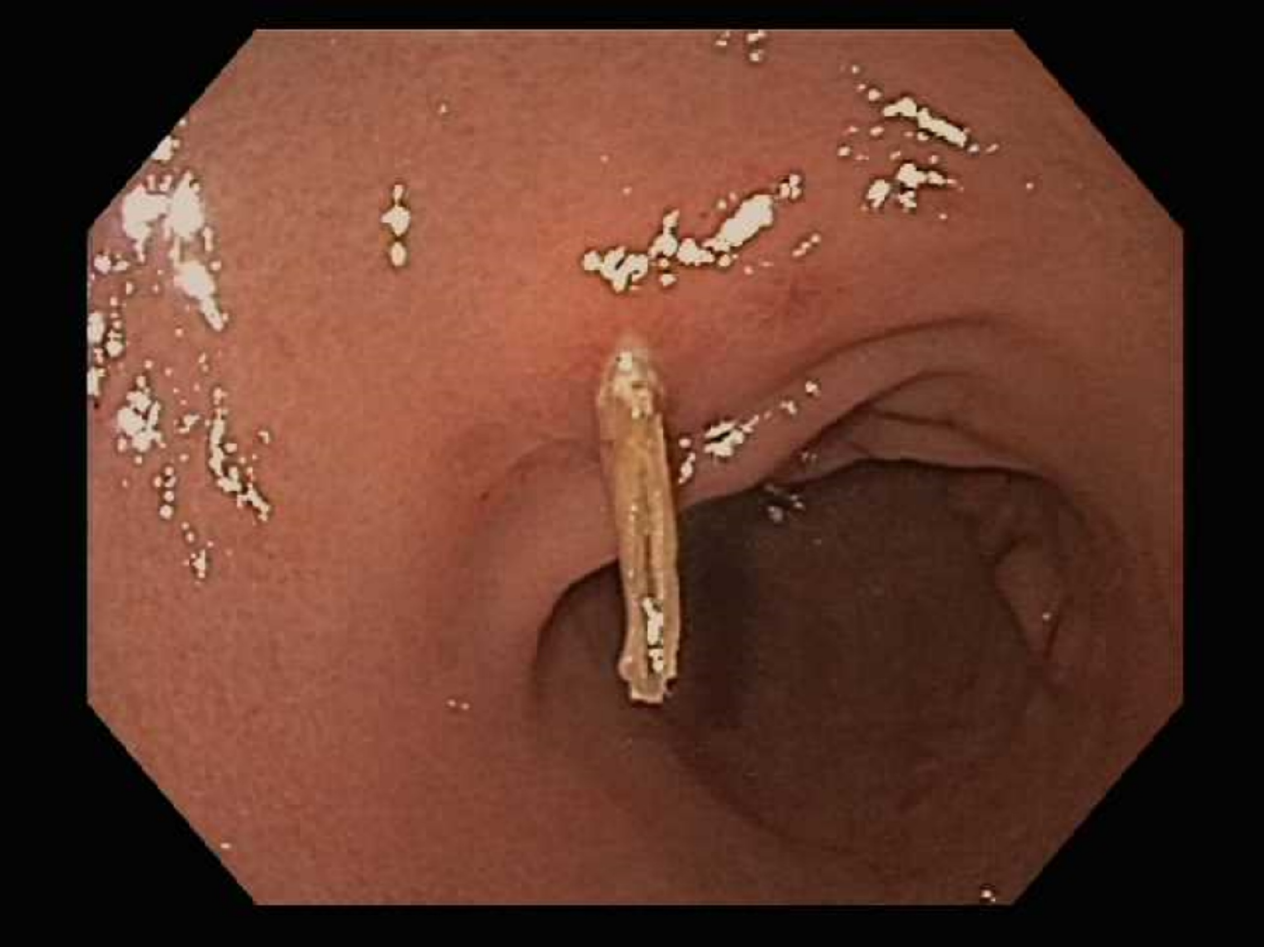
Endoscopy revealing wooden toothpick perforating the gastric antrum

**Figure 3:**
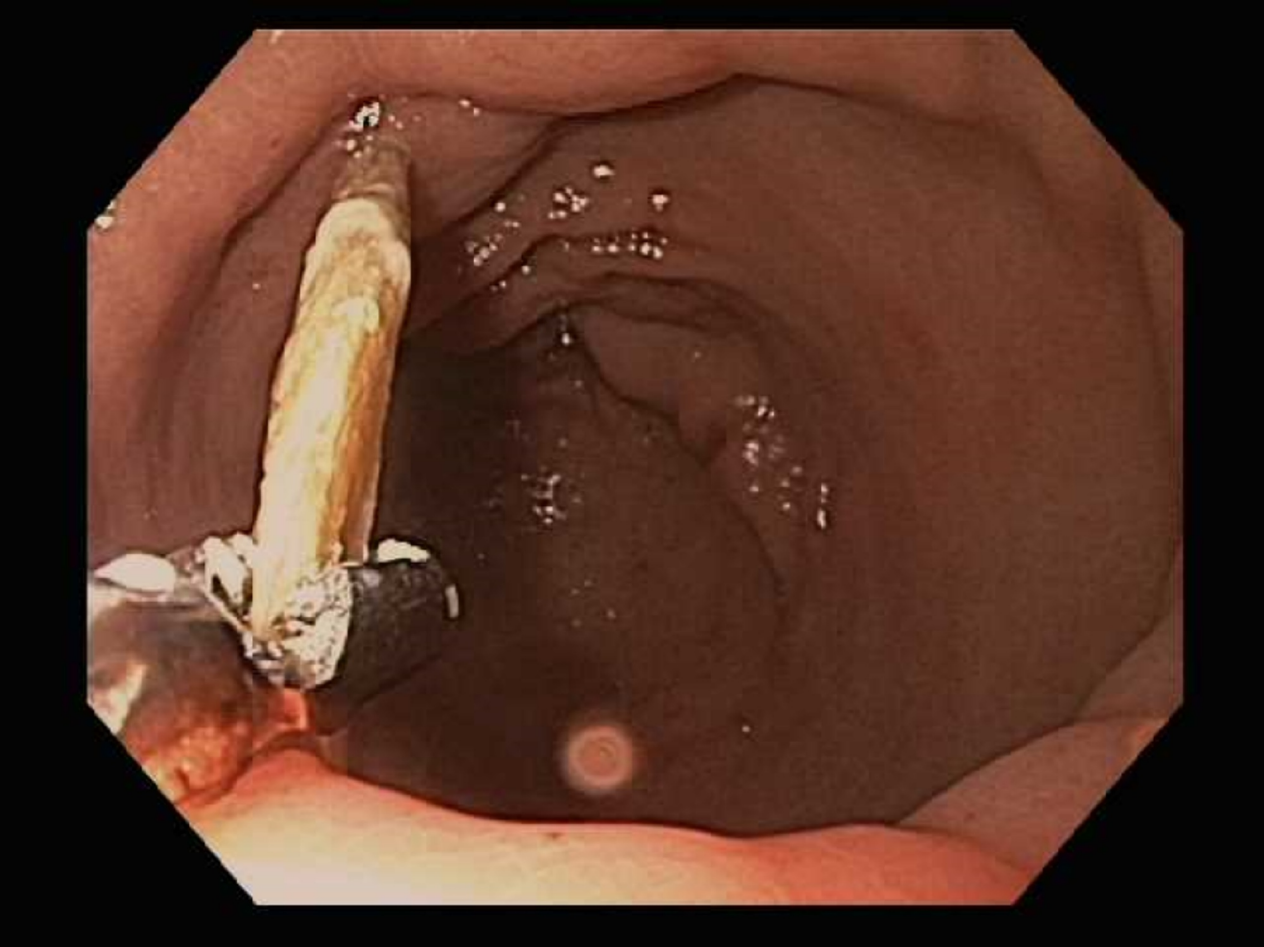
Endoscopic grasping of wooden toothpick with Raptor Grasping Device

**Figure 4:**
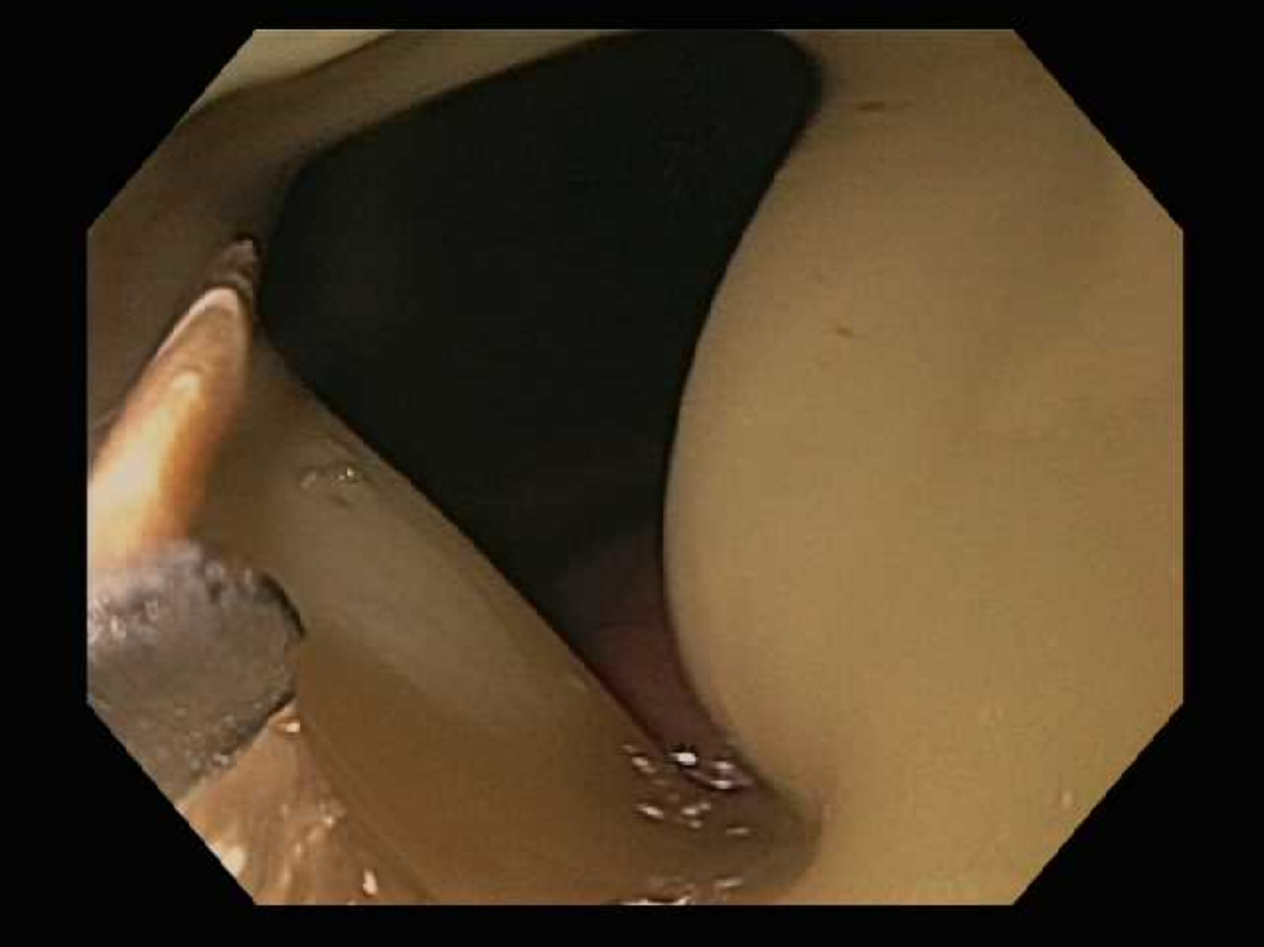
Endoscopic grasping of wooden toothpick within foreign body hood protector

**Figure 5:**
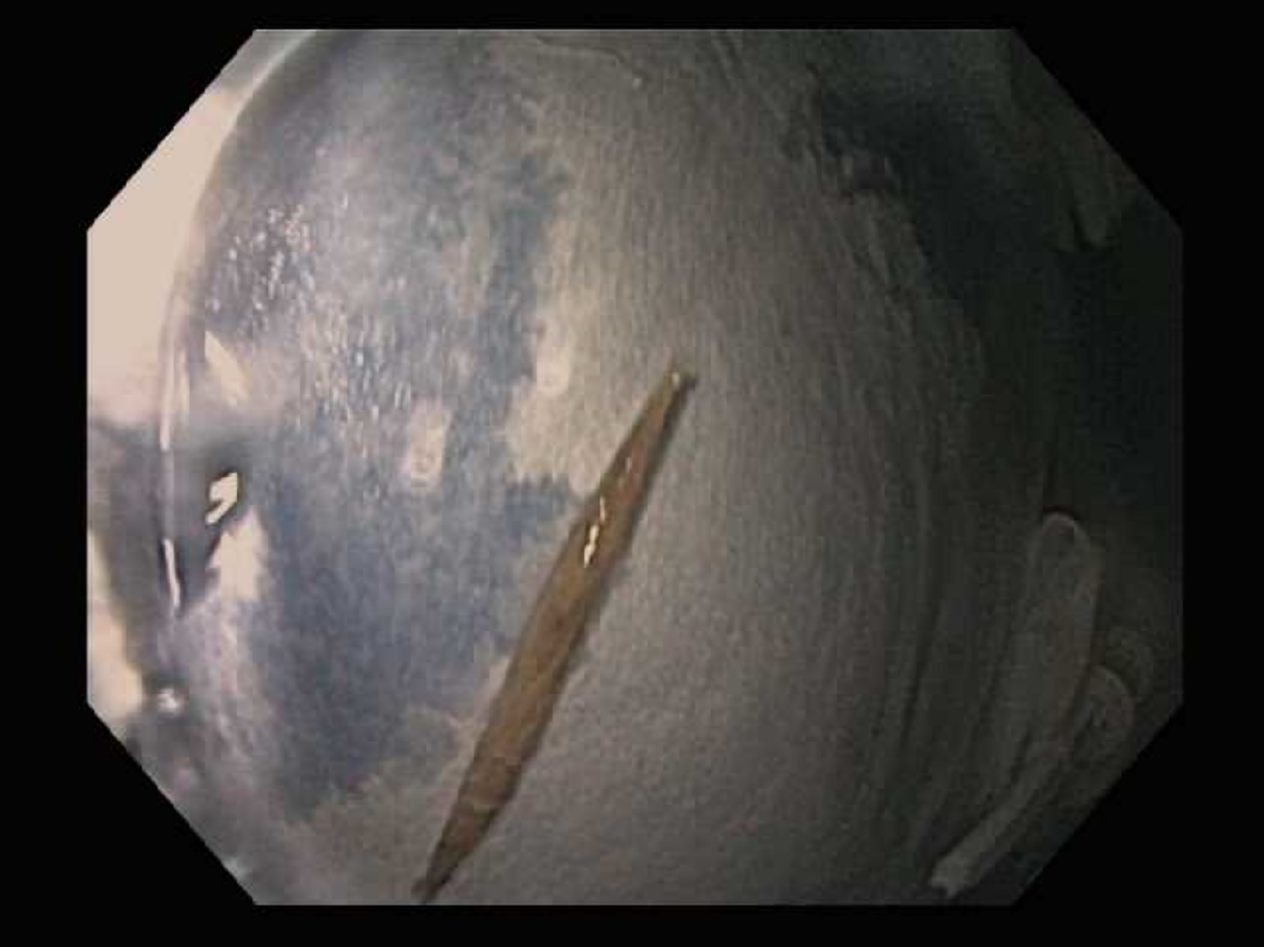
Full wooden toothpick removed from the patient

**Figure 6:**
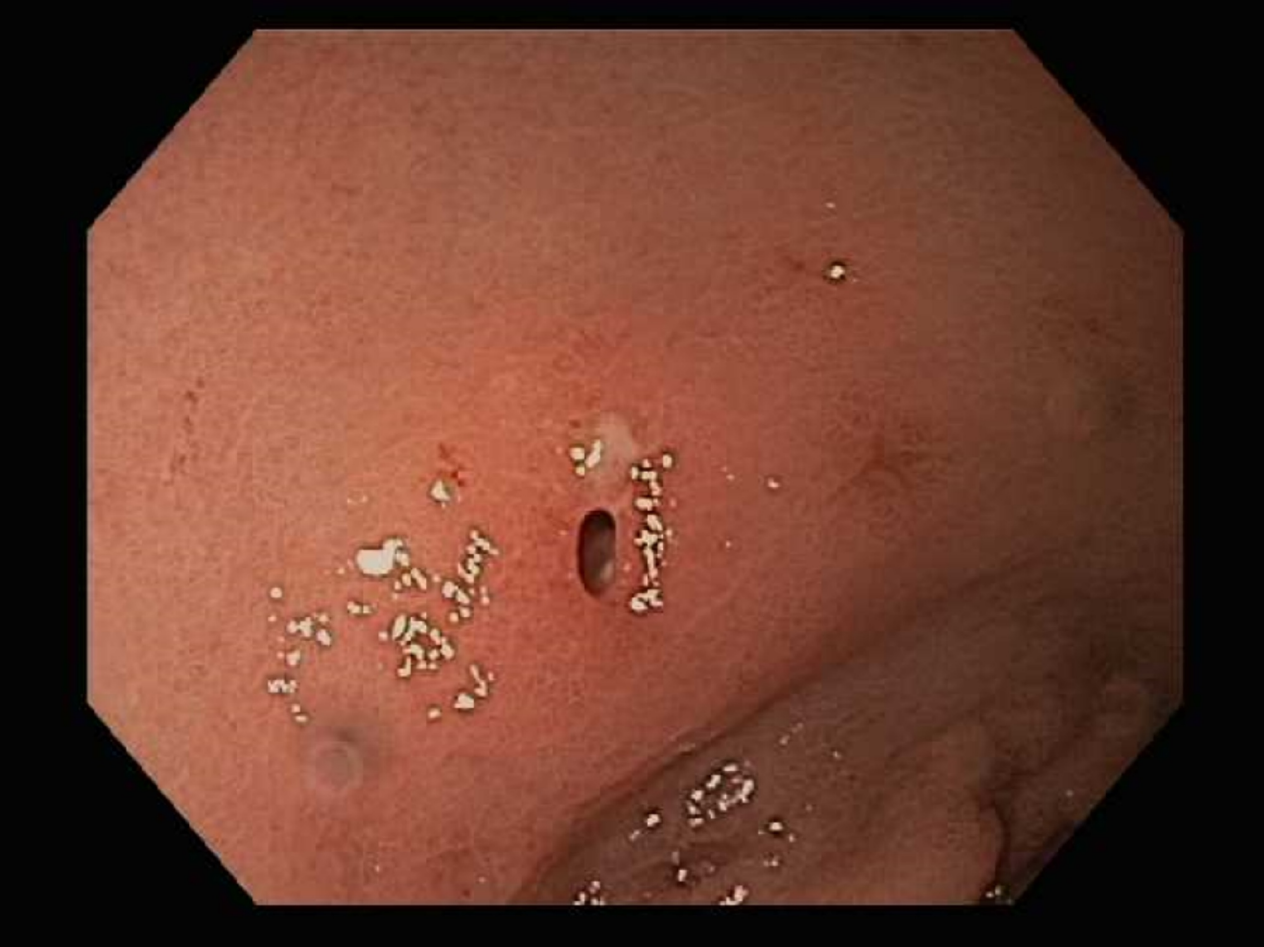
A 2 mm perforation site after removal of wooden toothpick

**Figure 7:**
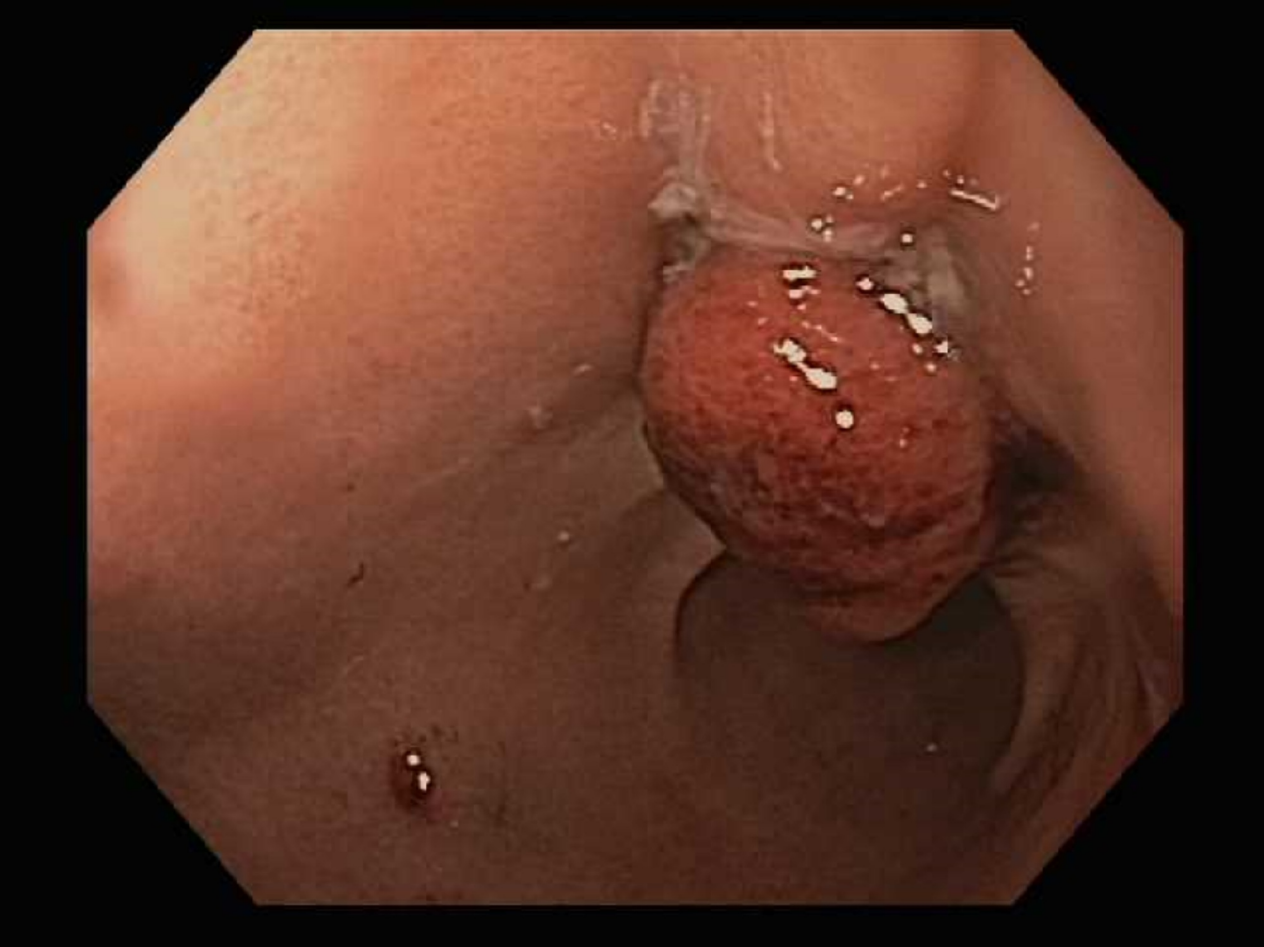
Endoscopic image of successful OTSC closure of perforation site OTSC, over-the-scope clip

Patient underwent an upper endoscopy revealing a toothpick in the gastric antrum perforating through the wall of the stomach (Figure 2). Endoscopic removal was successfully achieved using a Raptor Grasping Device (Steris, Mentor, OH, USA) with a foreign body hood protector (Figures 3, 4). Following removal of the toothpick, a full-thickness defect was found at the puncture site in the gastric antrum measuring 2 mm in diameter (Figures 5, 6). Endoscopic closure of the defect site was successfully achieved with an over-the-scope Padlock clip (Figure 7).

Post-procedurally, the patient recalled eating tacos three days prior with fragments of toothpicks in her food. Accidental ingestion caused gastric perforation with the sharp tip of the toothpick. The patient had an uneventful recovery and was discharged home.

**Figure 8:**
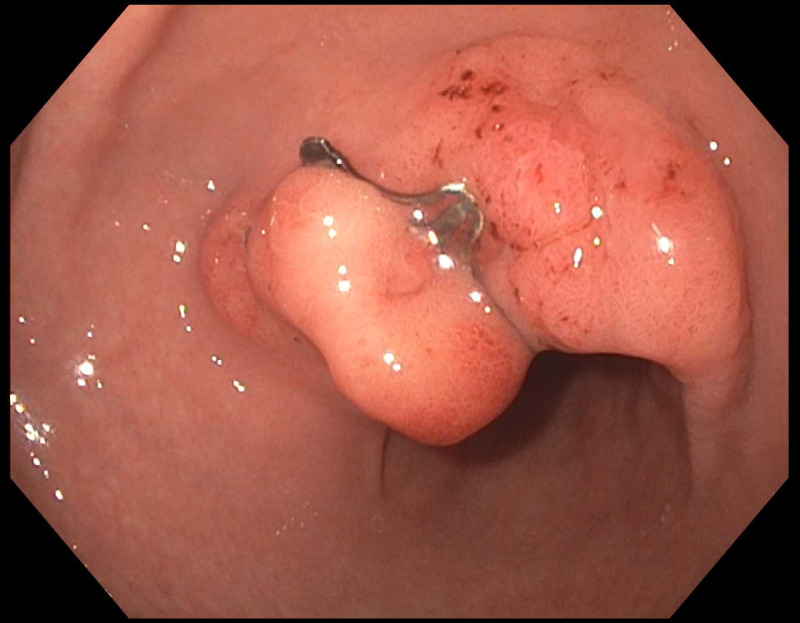
Endoscopic image of healed full-thickness defect with intact Padlock clip two years later

**Figure 9:**
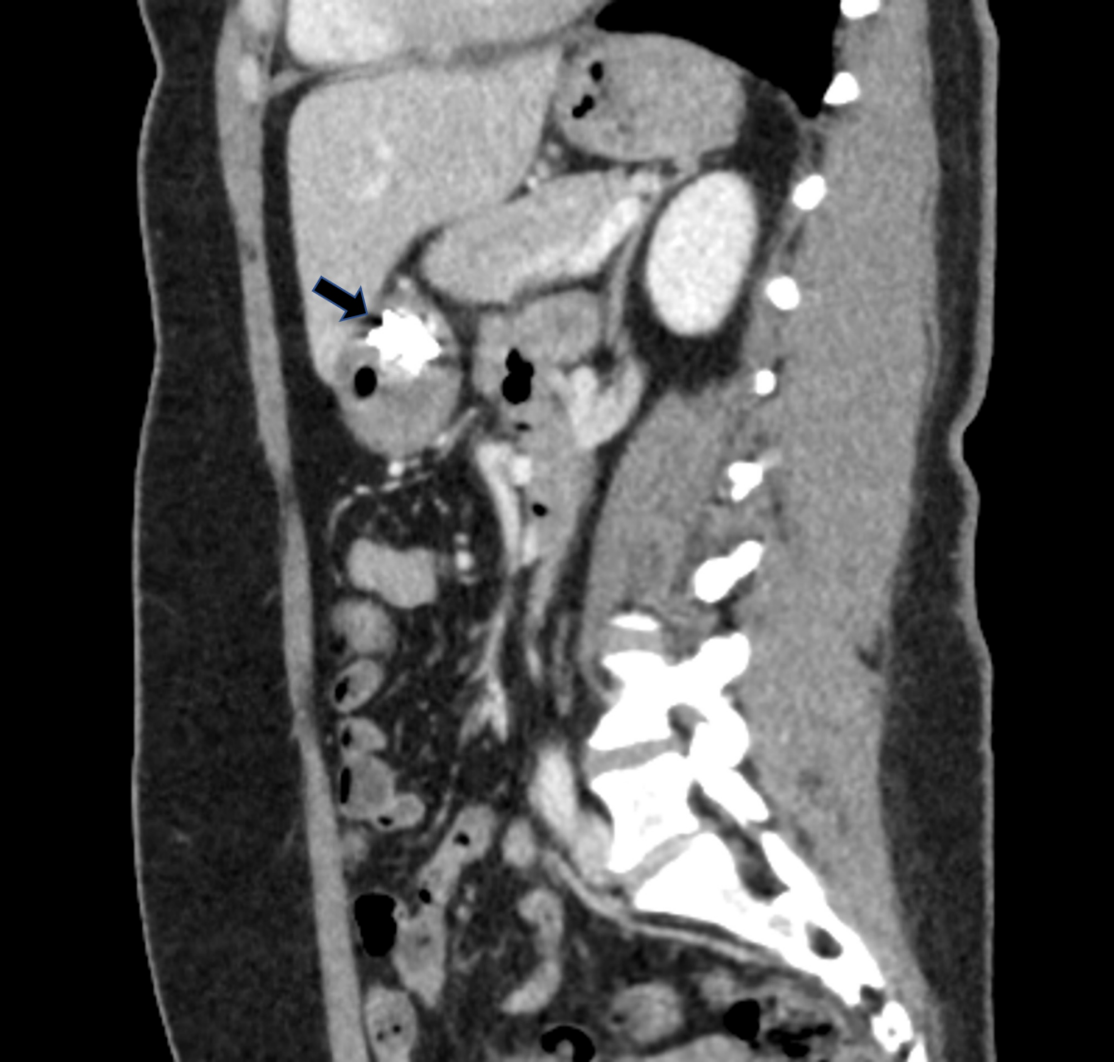
Abdominal CT scan revealing intact Padlock clip two years later

A follow-up endoscopy two years later revealed complete healing of the full-thickness defect with an intact Padlock clip (Figure 8). An abdominal CT scan at that time demonstrated complete closure by the intact Padlock clip without any abnormalities (Figure 9).

## Discussion

Our unique case highlights the importance of early endoscopic recognition and closure of a sharp foreign body induced gastric luminal perforation using an over-the-scope Padlock clip avoiding the need for major surgery. It was felt that an OTSC would be the preferred method of closure, as opposed to the use of an endoclip or endoscopic suturing, given the ability to achieve full-thickness closure, not requiring a change of endoscope, ease of use, and rapid deployment. Although emergent surgery had been the standard of care for foreign body removal, surgical interventions may be associated with higher morbidity and mortality than endoscopic therapies. In a retrospective review of 136 case reports, 49% of the ingested toothpicks were removed via surgical laparotomy and were associated with higher mortality compared to endoscopic removal. The overall mortality in these cases was 9.6%; however, those successfully treated endoscopically had a mortality of 0% (p = 0.011) [1]. Furthermore, a previous case report has illustrated the successful endoscopic removal and closure of toothpick induced colonic perforation utilizing an OTSC [9].

Endoscopic closure of luminal perforations using an OTSC device can be achieved with similar efficacy to a surgical intervention. In an ex vivo porcine stomach experiment, the acute strength of OTSC closures of gastric incisions were non-inferior compared with the gold standard of hand surgical suturing [7,10]. The successful use of OTSC closure of gastrointestinal perforations has been previously illustrated in the literature. In a large cohort of patients (n = 72) with iatrogenic perforations treated with OTSC, the clinical success rate was 86.1% [4,11]. In a study investigating the effectiveness of OTSC in the treatment of perforated peptic ulcers with a diameter of <15 mm, a technical and clinical success of 100% was achieved [4,12]. Utilizing OTSCs should be considered as a viable alternative to surgical repair of gastrointestinal perforations. 

## Conclusions

Early endoscopic recognition and removal of sharp foreign objects can help prevent the need for surgical interventions. Successful endoscopic closure of luminal perforations by OTSC devices can be achieved and should be considered in defects previously considered only amenable to surgical repair. Endoscopic treatment of gastrointestinal perforations has shown to decrease the morbidity and mortality associated with more invasive surgical procedures.
